# Ultrasound as a method to evaluate the distribution of abdominal fat in obese prepubertal children and the relationship between abdominal fat and metabolic alterations

**DOI:** 10.1590/0100-3984.2016.0230

**Published:** 2018

**Authors:** Arine Santos Peçanha, Alexandra Maria Monteiro, Fernanda Mussi Gazolla, Isabel Rey Madeira, Maria Alice Neves Bordallo, Cecilia N. Miranda Carvalho, Luciana Tricai Cavalini

**Affiliations:** 1 Universidade do Estado do Rio de Janeiro (UERJ), Rio de Janeiro, RJ, Brazil.; 2 Hospital Universitário Pedro Ernesto da Universidade do Estado do Rio de Janeiro (HUPE-UERJ), Rio de Janeiro, RJ, Brazil.

**Keywords:** Children, Ultrasonography, Subcutaneous fat, Intra-abdominal fat, Criança, Ultrassonografia, Gordura subcutânea, Gordura intra-abdominal

## Abstract

Abstract

**Objective:**

To evaluate, using ultrasound, the distribution of abdominal fat in obese prepubertal children, as well as its possible correlation with metabolic changes due to obesity.

**Materials and Methods:**

This was a cross-sectional study of prepubescent children: 77 obese children (33 girls and 44 boys), with a mean age of 7.31 years; and 31 normal-weight children (17 girls and 14 boys), with a mean age of 7.32 years. In all of the children, abdominal wall thickness (AWT) and abdominal fat thickness (AFT) were measured by ultrasound. For the evaluation of the associated metabolic alterations, serum levels of glycemia, HDL cholesterol, triglycerides, and insulin were determined.

**Results:**

The obese children presented with greater abdominal fat, predominantly greater AWT, without a significant gender-related difference in AWT or AFT. The homeostasis model assessment of insulin resistance (HOMA-IR) showed a significant direct correlation with AWT and AFT.

**Conclusion:**

In obese prepubertal children, the AWT, as measured by ultrasound, was shown to be more closely related to the HOMA-IR than to the lipid metabolism or glycemia.

## Abstract (pt)

**Objetivo:**

Avaliar, em crianças pré-púberes obesas, a distribuição da gordura no abdome por meio da ultrassonografia e sua possível correlação com as alterações metabólicas decorrentes da obesidade.

**Materiais e Métodos:**

Estudo transversal em crianças pré-púberes, sendo 77 obesas (33 meninas e 44 meninos) com média de idade de 7,31 anos e 31 eutróficas (17 meninas e 14 meninos) com média de idade de 7,32 anos. Em todas as crianças foram medidas a espessura da parede abdominal (EPA) e a espessura da gordura intra-abdominal (EIA), pela ultrassonografia. Para a avaliação das alterações metabólicas associadas, foi realizada dosagem sérica de glicemia, HDL-colesterol, triglicerídeos e insulina.

**Resultados:**

Observou-se que as crianças obesas apresentaram aumento da gordura abdominal, com predomínio da EPA e sem diferença significativa da EIA e a EPA em relação ao gênero. Foi encontrada associação direta estatisticamente significante entre o índice *homeostatic model assessment for insulin resistance* (HOMA-IR) e EPA e EIA.

**Conclusão:**

A EPA em pré-púberes obesos, medida pela ultrassonografia, demonstrou ter maior relação com o HOMA-IR, determinante de resistência insulínica, em relação ao metabolismo lipídico e à glicemia.

## INTRODUCTION

Childhood obesity is considered a public health problem worldwide^(1)^, being the most common cause of insulin resistance, as well as provoking other metabolic alterations^(2,3)^. In this context, the accumulation of fat in the abdomen and central distribution of that fat have been described as major risk factors for metabolic alterations and cardiovascular diseases^(4,5)^. Various imaging methods have been used in the evaluation of the distribution of abdominal fat^(6-11)^, such as computed tomography, magnetic resonance imaging, and ultrasound, the last being the most rapid method, being easily accepted, and not employing ionizing radiation, which is of great importance, especially in childhood^(12)^.

The purpose of this study was to use ultrasound as a method to evaluate the distribution of abdominal fat components and to correlate that distribution with the main metabolic alterations in obese prepubertal children.

## MATERIALS AND METHODS

This was a cross-sectional observational study conducted at the Pedro Ernesto University Hospital of the State University of Rio de Janeiro, including 77 children identified as being prepubertal according to the Tanner stage and classified as obese because they all had a body mass index at or above the 97th percentile^(13)^. The study group comprised 33 girls and 44 boys, with a mean age of 7.31 years (range, 5-11 years); none of the children had any associated diseases, and none had previously undergone a therapeutic intervention for weight loss. As a control group, we also included 31 prepubertal normal-weight children (17 girls and 14 boys) with a mean age of 7.32 years.

**Figure 1:**
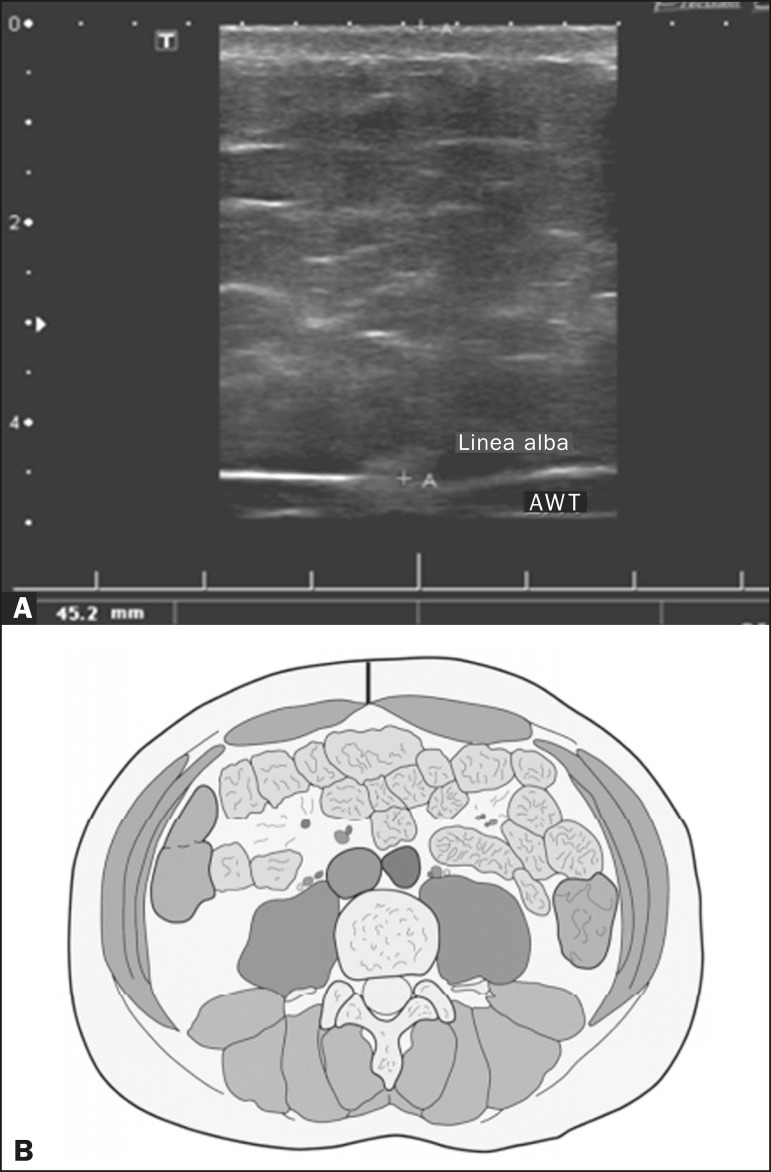
**A:** Ultrasound of the abdomen, in an axial view, approximately 2 cm below the emergence of the superior mesenteric artery. The markers (+) show the limits determined by the skin and the anterior face of the linea alba. **B:** Schematic diagram.

**Figure 2:**
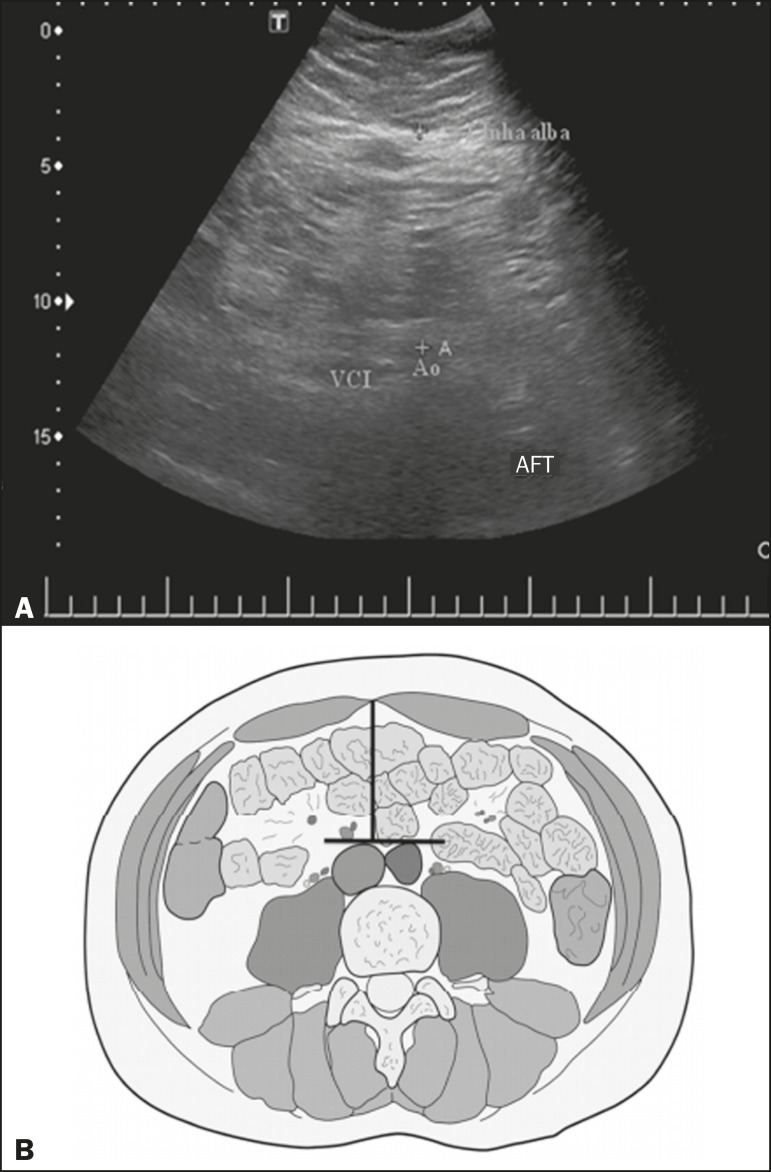
**A:** Ultrasound of the abdomen, in an axial view, approximately 2 cm below the emergence of the superior mesenteric artery. The markers (+) show the limits determined by the posterior aspect of the linea alba and the anterior wall of the aorta (Ao). **B:** Schematic diagram. VCI, inferior vena cava.

Ultrasound was performed by two physicians, each with more than 10 years of experience, in a double-blind manner. The subject was placed in the supine position, the transducer was situated approximately 2 cm above the umbilicus, and all measurements were taken in the axial plane, without any pressure of the transducer on the abdomen. The abdominal wall thickness (AWT) was defined as the distance between the skin and the anterior aspect of the linea alba (Figure 1). The abdominal fat thickness (AFT) was defined as the distance between the posterior aspect of the linea alba and the anterior wall of the aorta (Figure 2). We used an Aplio XG ultrasound device (SSA-790A; Toshiba, Tokyo, Japan) with a 3.5 MHz convex transducer for AFT measurement and a 12 MHz linear transducer for AWT measurement. No preparation was required prior to the examination. The mean examination time was less than 10 min, and the procedure was well tolerated by all of the patients.

In order to evaluate the metabolic alterations, we calculated fasting serum levels of high-density lipoprotein (HDL), triglycerides, glucose, and insulin, as well as the *homeostatic model assessment of insulin resistance* (HOMA-IR) index, which was determined by multiplying the value obtained for glucose (mmol/L) by that obtained for insulin (µIU/mL) and dividing the result by 22.5. The HOMA-IR cut-off point for normality was defined as 2.5^(14)^.

The data were stored in Excel for Windows version 8.0 (Microsoft Corp.) and analyzed by the R programming language, versions 3.1.0 and 3.1.1 (The R Foundation for Statistical Computing, Vienna, Austria). The continuous variables studied were HDL, triglycerides, fasting glycemia, AFT (in mm), and AWT (in mm). The categorical variables were gender, HOMA-IR status, HDL status, hypertriglyceridemia status, and glucose intolerance status.

Continuous variables were expressed as median ± standard deviation. The Wilcoxon test was used in order to compare the medians, and the chi-square test was used in order to evaluate possible AFT and AWT cut-off points that showed the strongest associations with the metabolic alterations evaluated. Multivariate linear regression models were adjusted for the parameters AFT and AWT, which were considered dependent variables. The independent variables included in the initial model were fasting glycemia, HDL cholesterol, triglycerides, HOMA-IR, age, and gender. In all statistical tests, a level of confidence of 95% (*p* ≤ 0.05) was adopted.

## RESULTS

In the obese prepubertal children evaluated, the median AFT was 41.40 ± 11.51 mm and the median AWT was 19.80 ± 6.74 mm, compared with 24.40 ± 8.36 mm and 5.80 ±2.12 mm, respectively, in the control (normal-weight) group. We identified no differences related to gender in either group.

Regarding the metabolic alterations, 23 children had an altered HOMA-IR index, 50 had a decreased HDL level, four had glucose intolerance, and 13 had hypertriglyceridemia. Twenty-two children presented with two or more of those alterations.

The median AFT and AWT values were compared between the groups with and without the metabolic alterations, which were analyzed individually (Table 1). On the basis of that analysis, we found that AFT and AWT both correlated significantly with an altered HOMA-IR index (*p* < 0.031 and *p* < 0.001, respectively); only AWT correlated significantly with hypertriglyceridemia (*p* < 0.038).

**Table 1:** Comparison of AFT and AWT in relation to the HOMA-IR index, HDL cholesterol, glucose intolerance, and hypertriglyceridemia.

	AFT				AWT		
	Normal	Altered	*P*		Normal	Altered	*P*
HOMA-IR	39.10	46.70	0.031*		18.05	22.70	< 0.001*
HDL	40.00	41.50	0.699		17.70	20.55	0.063
Glucose intolerance	41.40	40.95	0.615		19.60	21.70	0.342
Hypertriglyceridemia	40.70	43.50	0.488		19.00	20.80	0.038*

* Statistically significant difference (*p* < 0.05).

In the multivariate linear regression models used for the AFT and AWT parameters, the independent variables were fasting glycemia, HDL cholesterol, triglycerides, HOMA-IR, age, and gender. In that model, there was no correlation between hypertriglyceridemia and AWT. We found that the HOMA-IR index correlated directly with AFT and AWT. For each unit increase in the HOMA-IR index, there was an increase of 1.313 mm in the AFT (*p* < 0.0262) and an increase of 1.021 mm in the AWT (*p* < 0.0027), as shown in Tables 2 and 3, respectively. However, the correlation between the AWT and the HOMA-IR index retained its statistical significance after the insertion of the variables gender and age into the model (Table 3), whereas that between the AFT and the HOMA-IR index did not (Table 2).

**Table 2:** Result of multivariate linear regression analysis considering AFT as the outcome variable.

	Model 1			Model 2	
Variable	Coefficient β	*P*		Coefficient β	*P*
HOMA-IR	1.313	0.0262*		1.0921	0.0833
Age	-			0.9187	0.3881
Gender	-			3.6169	0.1644

* Statistically significant difference (*p* < 0.05).

**Table 3:** Result of multivariate linear regression analysis considering AWT as the outcome variable.

	Model 1			Model 2	
Variable	Coefficient β	*P*		Coefficient β	*P*
HOMA-IR	1.021	0.0027*		0.7852	0.0296
Age	-			1.0656	0.0809
Gender	-			–0.7094	0.6297

* Statistically significant difference (*p* < 0.05).

## DISCUSSION

Although childhood obesity is considered a global epidemic, there have been few studies correlating serum metabolic alterations with measurements of abdominal fat distribution by imaging methods, especially in prepubertal children. In this age group, there is still no action of sexual steroids that may interfere with metabolic evaluation^(15,16)^. In the present study, we used ultrasound as a method to evaluate abdominal fat distribution in correlation with fasting glycemia, HDL cholesterol, triglycerides, and the HOMA-IR index in 77 obese prepubertal children, showing that only the HOMA-IR index correlated significantly with AFT and AWT, notably with the latter.

Regarding the lipid metabolism, Semiz et al.^(17)^ also found no relationship with AFT, whereas other authors have identified a positive correlation between AFT and hypertriglyceridemia^(18,19)^. In addition, Jung et al.^(19)^ found a negative correlation between AFT and HDL cholesterol. However, in all of those studies^(17-19)^, the study sample included prepubertal and pubertal children who were older than those evaluated in our study, which could have influenced the findings, given that the literature shows that alterations in lipid metabolism can be influenced by the degree of sexual maturity^(20)^; that is, by the influence of sex hormones at the different stages of sexual maturation. None of the studies cited evaluated the AWT^(17-19,20)^.

Various studies have evaluated fasting glycemia in correlation with the distribution of abdominal fat^(17,21)^. None of those studies have demonstrated a statistically significant correlation between the two, which is in keeping with our findings.

The HOMA-IR index reflects insulin resistance^(22)^. Therefore, it is a relevant marker of altered metabolism, as has been described in various studies^(17-19,21)^, notably those conducted by Reinehr et al.^(18)^ and Reyes et al.^(21)^, who also identified a correlation between AFT and the HOMA-IR index.

It was not possible to correlate our findings in relation to AWT with those of some previous studies^(18,19,21)^, in which AWT was not measured.

In agreement with our findings, Semiz et al.^(17)^, demonstrated that AWT correlated significantly with metabolic risk factors but found no such correlation for AFT. Those authors evaluated 33 children with mean age of 12 ± 2.7 years, although they did not distinguish between pubertal and prepubertal children, which might have been the specific cause of their findings being similar to ours, due to a possible predominance of the prepubertal group over the pubertal group. In general, in the distribution of adiposity in children, regardless of gender, a predominance of the subcutaneous component has been observed^(23)^, as it was in our obese prepubertal group. In obese children, that predominance seems to correlate positively with an alteration in the HOMA-IR index, suggesting some kind of effect on the development of insulin resistance, which is different from what is described for the adult population^(17)^. Therefore, the fact that most of the children in our sample showed a predominance of accumulation of fat in the abdominal wall indicates that it could be a factor related to insulin resistance. There is a need for further studies, considering puberty separately from chronological age, so that correlations can be established between the metabolic changes related to childhood obesity and the distribution of abdominal fat.

## CONCLUSION

In obese prepubertal children, the AWT, as measured by ultrasound, appears to have a stronger relationship with the HOMA-IR index, a determinant of insulin resistance, than with the lipid metabolism or glycemia.
